# Clinical presentation and outcomes in children with retinoblastoma managed at the Uganda Cancer Institute

**DOI:** 10.1155/2022/8817215

**Published:** 2022-03-08

**Authors:** Abubakar Kalinaki, Haruna Muwonge, Joyce Balagadde-Kambugu, Yusuf Mulumba, Jacob Ntende, Grace Ssali, Lydia Nakiyingi, Damalie Nakanjako, Caroline Nalukenge, Anne M. Ampaire

**Affiliations:** ^1^Department of Ophthalmology, College of Health Sciences, Makerere University, P. O. Box 7072, Kampala, Uganda; ^2^Habib Medical School, Islamic University in Uganda, Uganda; ^3^Department of Physiology, College of Health Sciences, Makerere University, P. O. Box 7072, Kampala, Uganda; ^4^Uganda Cancer Institute, P.O.Box, 3935, Kampala, Uganda; ^5^Department of Medicine, College of Health Sciences, Makerere University, P. O. Box 7072, Kampala, Uganda

## Abstract

**Background:**

The majority of patients with retinoblastoma, the most common intraocular cancer of childhood, are found in low-and middle-income countries (LMICs), with leukocoria being the most common initial presenting sign and indication for referral. Findings from the current study serve to augment earlier findings on the clinical presentation and outcomes of children with retinoblastoma in Uganda.

**Methods:**

This was a retrospective study in which we reviewed records of children admitted with a diagnosis of retinoblastoma at the Uganda Cancer Institute from January 2009 to February 2020. From the electronic database, using admission numbers, files were retrieved. Patient information was recorded in a data extraction tool.

**Results:**

A total of 90 retinoblastoma patients were studied, with a mean age at the first Uganda Cancer Institute (UCI) presentation of 36.7 months. There were more males (57.8%) than females, with a male to female ratio of 1.37 : 1. The majority (54.4%) had retinoblastoma treatment prior to UCI admission. The most common presenting symptoms were leukocoria (85.6%), eye reddening (64.4%), and eye swelling (63.3%). At 3 years of follow-up after index admission at UCI, 36.7% of the patients had died, 41.1% were alive, and 22.2% had been lost to follow-up. The median 3-year survival for children with retinoblastoma in our study was 2.18 years. Significant predictors of survival in the multivariate analysis were follow-up duration ($\underset{¯}{P}<0.001$), features of metastatic spread (*P* = 0.001), history of eye swelling (*P* = 0.012), and bilateral enucleation (*P* = 0.011).

**Conclusions:**

The majority of children who presented to the Uganda Cancer Institute were referred with advanced retinoblastoma, and there was a high mortality rate. Retinoblastoma management requires a multidisciplinary team that should include paediatric ophthalmologists, paediatric oncologists, ocular oncologists, radiation oncologists, and nurses.

## 1. Introduction

Retinoblastoma is a primary intraocular childhood malignancy arising from the embryonal cells of the retina and the most common primary intraocular malignancy of childhood, with over 90% of cases diagnosed before the age of five [1–3]. The retinoblastoma incidence in the USA is 1 in 15,000–20,000 live births, with 60% of cases presenting with unilateral disease [4]. It is estimated that 250 to 300 new cases in the United States of America and approximately 8,000 to 9,000 cases worldwide of retinoblastoma are diagnosed every year [3, 5]. However, the annual incidence of retinoblastoma is the same across geographical regions, and the annual prevalence of retinoblastoma is higher in Africa than in higher income countries [3, 6].

Studies show that over 95% of children with retinoblastoma in the United States and other developed nations survive their malignancy, whereas approximately 50% survive worldwide [5]. In low-income countries, patients with retinoblastoma are diagnosed at a median age of 30.5 months, with 49.1% of these presenting with extraocular disease and 18.9% having metastatic disease [3]. In contrast, patients from high-income countries were diagnosed at a median age of 14.1 months, with 98.5% of these having intraocular retinoblastoma and only 0.3% exhibiting metastatic disease [3]. Consequently, a higher median age at diagnosis extended lag time from first presentation until patients reach a retinoblastoma treatment centre, and advanced disease at index diagnosis implies that retinoblastoma patients from low-income countries have poor survival rates compared to retinoblastoma patients from high-income countries [3, 7].

In a retrospective study performed on retinoblastoma children in India, leukocoria was the most common presenting symptom, with 21 months being the median age at which first signs were observed by parents, with one-third of patients presenting with bilateral disease at first diagnosis [8]. In this study, age at presentation, lag period, and stage of disease were found to be associated with poor survival outcomes. In a retrospective study performed in the Democratic Republic of Congo, the average age at diagnosis of retinoblastoma patients was 32 months, with the first sign of disease noticed by parents at a mean age of 20 months, thereby resulting in a year of lost time before reaching hospital for diagnosis and treatment [9]. These figures are similar to those reported in an earlier Ugandan study by Waddell Keith and colleagues, where the median age at diagnosis was 33 months and 17.5 months for unilateral and bilateral disease, respectively [10].These delays in diagnosis were thought to contribute to the high mortality from retinoblastoma in Ugandan patients.

Retinoblastoma treatment has shifted towards increased utilization of chemotherapy and less radiotherapy, with surgical treatment remaining a predominant and relevant treatment modality. A study by Waddell et al. revealed a higher mortality rate among retinoblastoma children prior to the utilization of chemotherapy modalities, even in those with intraocular disease [10]. However, in a retrospective population-based cohort study in Taiwan, the survival outcome was significantly associated with enucleation intervention [11]. Intraocular retinoblastoma allows vision and eye salvage, whereas extraocular disease has nearly no possibility of vision salvage [8].

Before 2015, most of the children with or suspected to have retinoblastoma were referred for further treatment to Ruharo eye centre, a missionary hospital in western Uganda. Here, children with retinoblastoma who needed chemotherapy were treated with a full chemotherapy regimen (vincristine, etoposide, and cyclophosphamide). However, in the last 4 years, more of these children have been referred to the Uganda Cancer Institute (UCI), a regional centre of excellence in oncology facilities in East Africa. With the increasing number of children with retinoblastoma admitted and managed at the paediatric oncology unit of UCI, there was a need to study the pattern of clinical presentation, outcomes and predictors of survival among children with retinoblastoma.

This study aimed to describe the clinical presentation, outcome, and predictors of survival of children managed at UCI. The correlation between the clinical presentation and outcomes will help the health care team involved in the management of retinoblastoma patients to mobilize more resources and develop protocols that will improve patient outcomes.

## 2. Materials and Methods

### 2.1. Study Design and Setting

A retrospective review of records of children with retinoblastoma admitted to the Uganda Cancer Institute from 1^st^ of January 2009 to 28^th^ of February 2020. The Uganda Cancer Institute is a regional centre of excellence for oncology in East Africa. The inclusion criteria included children below 18 years with retinoblastoma diagnosis admitted and files opened at UCI between January 2009 and February 2020. We excluded 7 patients whose files/charts were missing important information, and whose caretakers could not be contacted. A total of 90 patients with 115 eyes were enrolled in the study. The following data were collected from the medical records: age at diagnosis, sex, clinical signs and symptoms, laterality, treatment (chemotherapy, surgery, and radiotherapy), family history, and vital status (alive or dead). Follow-up information on study participants was also extracted from the charts, and missing information (especially vital status) was obtained through telephone interviews with patients' caretakers. The primary endpoint in this study was 3-year survival, and the secondary endpoint was the ocular outcome (number of salvaged eyes and presence of vision). This study was approved by the School of Medicine Research Ethics Committee (#2020-031).

The diagnosis of retinoblastoma was clinically and/or histopathologically made by ophthalmologists, pediatric oncologists, and pathologists. Intraocular disease was classified using the International Intraocular Retinoblastoma Classification. Staging was performed at the UCI to determine the treatment of choice for each participant.

Treatment consisted of one or a combination of the following treatment modalities: chemotherapy, surgery (unilateral or bilateral enucleation), and external beam radiotherapy.

### 2.2. Data Management and Analysis

The extracted data were entered into an electronic database using Epidata 4.2 by the double entry method. The database was checked and cleaned for consistency. The cleaned data were exported into STATA 17 for analysis. Continuous variables such as age and lag time were summarized as the mean ± standard deviation. Categorical variables such as sex, clinical signs and symptoms, laterality, and vital signs were summarized in proportions or frequencies and presented as tables or graphs. The time-specific survival rate (3-year survival) with a 95% confidence interval (CI) was estimated by the Kaplan–Meier method and assessed by a log-rank test. Three-year survival was defined as the period of follow-up of 3 years from the date of index admission at UCI. Cox regression analysis was used to assess the predictors of outcome and presented as hazard ratios with their 95% confidence intervals at both bivariate and multivariate levels. A *P* value <0.2 was considered at bivariate analysis, and all variables that had *P* < 0.2 were entered at multivariate Cox regression analysis. A *P* value <0.05 was considered significant.

## 3. Results

A total of 97 charts with retinoblastoma diagnoses were retrieved from the records department of the Uganda Cancer Institute, and 90 charts that had confirmed retinoblastoma diagnoses were studied. There was a mean age of 33.3 months (SD 25.3) for bilateral retinoblastoma and 38 months (SD 25.6) for unilateral retinoblastoma, with an overall mean age at first UCI presentation of 36.7 months (SD 25.5). There were more males than females, with a male to female ratio of 1.37 : 1. The majority of study subjects (36.7%, *n* = 33) came from the central region of the country, and 13.3% (*n* = 12) of children were nationals of neighbouring countries (Table 1).

Leukocoria was the most common retinoblastoma sign (77, 85.6%) reported by parents prior to the first UCI visit, followed by eye reddening (58, 64.4%) and eye swelling (57, 63.3%). There was a family history of retinoblastoma among first-degree relatives in 4.4% of children. The majority of patients had prior retinoblastoma treatment at eye departments of tertiary hospitals before UCI admission, with 46.7% having had enucleation of at least one eye, as shown in Table 1. A significant number (37, 41%) of children had a lag time to UCI admission of at least 12 months (Table 1).

Using the international intraocular retinoblastoma classification scheme (IIRC), most eyes in our study presented with advanced disease of IIRC groups D (20%) and E (53%). Twenty eyes (17%) had no record of their IIRC classification (Table 2).

Sixty-five patients (72.3%) presented with unilateral retinoblastoma, and proptosis was the most common (45%, *n* = 41) presenting sign, including children with recurrent orbital retinoblastoma, followed by leukocoria 36 (40%).

The majority (55, 97.8%) of children with unilateral retinoblastoma whose IIRC staging were performed had Group E (81.8%) classification, followed by D (18.1%) classification**(**Table 2**).**

**Figure 1:**
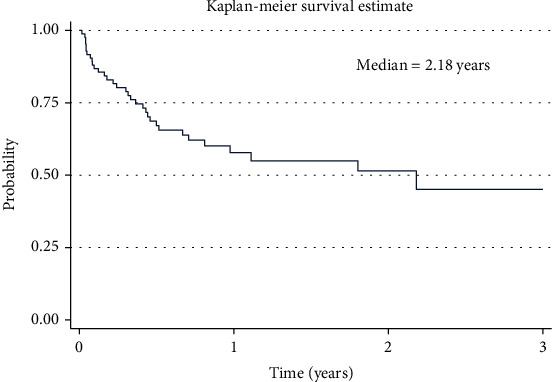
Graph showing the 3-year survival of children with retinoblastoma from the time of admission to the Uganda Cancer Institute.

At the 3-year follow-up after admission at UCI with a diagnosis of retinoblastoma, 41.1% of the patients were still alive, and 36.7% of the patients had died. Furthermore, 54.4% and 3.3% had unilateral and bilateral eye loss, respectively **(**Table 3**).** The median survival time in years for children with retinoblastoma in our study was 2.18 years. **(**Figure 1).

**Figure 2:**
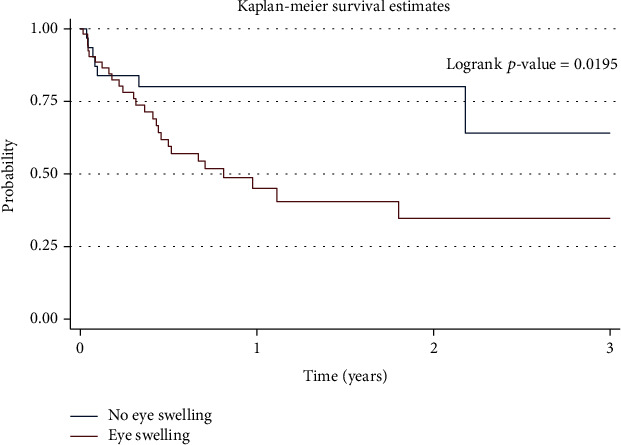
Graph showing the 3-year survival of children with retinoblastoma by history of eye swelling.

**Figure 3:**
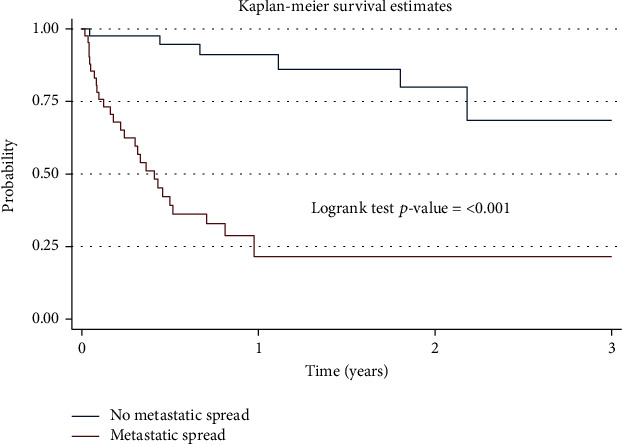
Graph showing the 3-year survival of children with retinoblastoma by metastasis.

The factors that were statistically significant predictors of mortality in the multivariate analysis were lag time between first sign and UCI visit, history of eye swelling (Figure 2), features of metastatic spread (Figure 3), and bilateral enucleation. Children with metastatic spread (AHR 8.93, CI 3.23–24.69, *P* = 0.001) were 8.9 times more likely to die than those without metastatic spread. Children who presented with a history of eye swelling prior to any retinoblastoma treatment (AHR 4.39, CI 1.38–14.01, *P* = 0.012)) were 4.4 times more likely to die than those who had no history of eye swelling. In addition, children who underwent bilateral enucleation (AHR 3.53, CI 1.33–9.36, *P* = 0.011) were more likely to die than those who did not undergo enucleation (Table 4).

## 4. Discussion

Charts for 90 children with retinoblastoma diagnosis were reviewed. There was a female to male ratio of 1 : 1.4. With the exception of a study in Iran, which showed a higher female to male ratio, the findings in our study were similar to those of other studies performed in India, Singapore, Eastern Nepal, Kinshasa (DRC), and Gezira-Sudan, which revealed a higher male to female ratio [8, 9, 12, 13]. The majority of the patients (95%) had their first presentation at UCI beyond 12 months of age, with 33% (30 children) at least 3 years old. This is because the largest percentage of children underwent retinoblastoma management prior to admission.

In our study, the overall mean age at the first UCI visit, 36.7 months (SD ± 25.5), was slightly higher than that from a study performed in Eastern Nepal [13]. Our study also revealed a lower difference in age at first UCI presentation between unilateral (38.0(SD ± 25.6) and bilateral (33.3(SD ± 25.3) retinoblastoma cases than that reported in other studies [14, 15]. The disparity in age at first presentation is because a significant number of children (*n* = 49, 54.4%) admitted at UCI had prior retinoblastoma management.

There was no record of IIRC classification for 12 (13.3%) children. This finding complicates the management and follow-up of children with retinoblastoma. This was found mostly in children who underwent enucleation prior to UCI admission. The majority of children had the worst affected eyes with Group E (*n* = 61, 67.8%) IIRC classification, followed by Group D (*n* = 16, 17.8%). The majority (*n* = 55, 97.8%) of children with unilateral Rb whose IIRC staging was performed had Group E (81.8%), followed by Group D (18.1%). This finding is comparable to other studies conducted in less developed countries [9, 10, 14–16].

Sixty-five (72.3%) children presented with unilateral retinoblastoma, whereas 25 (27.8%) presented with bilateral disease. These findings were similar to those of other studies, which showed higher unilateral retinoblastoma cases than bilateral retinoblastoma. For instance, studies conducted in India by Chawla et al. and in Kinshasa by Kazadi et al. found that 67.6% and 77.5% of children presented with unilateral retinoblastoma [8, 9].

In our study, the most common retinoblastoma sign that prompted parents to seek eye health attention was leukocoria, reported in 77 (85.6%) patients, followed by eye reddening in 58 (64.4%) and eye swelling in 57 (63.3%), all of which are features of advanced disease. Our findings were similar to those from other studies that revealed leukocoria as the most common sign noted by parents of children with retinoblastoma in India (83%), Singapore (70.6%), and the Democratic Republic of Congo (DRC) (67.5%) [8, 9, 13]. Eye reddening (58, 64.4%) and eye swelling (57, 63.3%) were the second most common signs noted by parents in our study. Proptosis was shown to be the second most common sign noticed by parents in studies in India (17%) and in the DRC (15%) [8, 9, 13]. The percentage of children with proptosis as one of the most common signs was higher in our study than in other studies. This shows that most children in our study sought their first medical attention with advanced disease.

At first presentation at the Uganda Cancer Institute, proptosis was the most common presenting sign reported in 41 (45.6%) children, followed by leukocoria reported in 36 (40%) children. This finding is similar to studies by Kazadi Lukusa et al. in the DRC, with 55% and 25% of children with retinoblastoma presenting with proptosis and leukocoria, respectively [9]. In addition, proptosis was the most common presenting sign (56%) at the National Cancer Institute in Gezira, followed by leukocoria (32%) [12].

A family history of retinoblastoma in first-degree relatives (parents, offspring, and siblings) was reported in 4.4% (*n* = 4) of children who were admitted at UCI. Our findings are consistent with findings from similar studies in Iran and Taiwan, where a family history of retinoblastoma was reported in 4.8% and 3.1% of patients, respectively (18, 19) In contrast, a family history of retinoblastoma was reported in 11.0% of children treated between 1981 and 2004 at a medical facility in Turkey [17]. The low percentage of children with a family history of retinoblastoma in our study is possibly due to a lack of knowledge about eye cancers in our community. In addition, patients do not survive to child bearing age.

The mean lag time between the first sign noted by parents and the first UCI visit was 11.2 months (SD 10.8), with a mean lag time of 14.5 months and 9.9 months for bilateral and unilateral retinoblastoma, respectively. A significant number of children (41.1%) had a longer lag time (beyond 12 months). This is because most children were managed at local eye units prior to referral to UCI. Consequently, lag time was a significant predictor of 3-year survival, as patients with retinoblastoma who reported to UCI more than 7 months after developing the first sign of retinoblastoma were more likely to die within 3-years compared to patients who presented within at least 6 months of developing the first sign of retinoblastoma (AHR 0.10, CI 0.04–0.25, *P* = 0.001).

In our study, the majority of children had the worst affected eye, with IIRC Group E 61 (67.8%), followed by Group D 16 (17.8%). Additionally, most children with unilateral Rb (*n* = 55, 97.8%) whose IIRC staging was performed had Group E staging (81.8%) followed by D staging (18.1%). The majority of children (63, 70%) had features of extraocular spread at diagnosis at the first UCI visit. These findings were similar to a study by Lim et al., which showed a higher percentage of children (88.6%) with Group E and D staging [18]. In a study by Waddell et al., nearly all diagnoses (*n* = 282) in the first affected eye were Group E or already had extraocular disease, with only 2 children having IIRC Group C eyes [10]. The lower percentage of findings in our study compared to that of Waddell et al. may be attributed to the awareness campaign for primary health care workers together with patient facilitation, which was recommended.

In our study, management included all treatment received by the children in the study prior to and after UCI admission. Forty-nine (54.4%) had received treatment prior to UCI admission, with 42 (46.7%) having undergone enucleation. At the last follow-up, an additional 10 (11.1%) children underwent enucleation after UCI admission.

Vision and globe salvage were attempted in 13 (11.3%) and 15 (13%) eyes, respectively. However, the only treatment option utilized was chemotherapy. The majority (68, 75.6%) of children received chemotherapy, 43 (47.8%) received neoadjuvant chemotherapy, and 18 (20%) underwent radiotherapy. Seventy-five (83.3%) out of the 90 patients admitted into care at UCI were initiated on prescribed treatment. The follow-up duration for the majority (59, 65.6%) of children was less than 6 months from the time of the first UCI visit. The utilization of chemotherapy for Rb treatment, alongside other treatment modalities, has been embraced at UCI for the majority of children, which corresponds with current treatment recommendations [19].

At the 3-year follow-up from the first UCI visit, 37 (41.1%) of all children in our study were alive, and 33 (36.7%) of children with retinoblastoma had died. The number of those lost to follow-up was 20 (22.2%). The 3-year survival observed in our study (41.1%) was significantly lower than that reported in Taiwan (64.41%), Turkey (89.6%), and Iran (94.8%) [17, 20, 21]. The significantly low 3-year survival in our study could be attributed to advanced disease at first UCI admission. Although enucleation was one of the most utilized treatment modalities in our study (57.8%, *n* = 52)), it was lower than that in studies in India by Chawla et al. (95.7%), in a country-wide study in Uganda by Waddell et al. (96%), and in Iran by Nabie et al. (95.7%) [8, 10, 16]. This finding can be explained by the increased utilization of neoadjuvant chemotherapy at UCI. Forty-nine (54.4%) and three (3.3%) of the children in our study had unilateral and bilateral enucleation, respectively. The lower percentage of enucleation in our study may be attributed to the noncompletion of prescribed treatment options, which was recorded in 61 (67.8%) children.

At 3 years of follow-up from first admission at UCI, 12 (13.3%) and 64 (71.1%) children in our study had bilateral and unilateral vision loss, respectively, including those who had enucleation in the affected eye(s). The significant number of children with vision loss in our study is attributed to the unavailability of vision salvage treatment modalities at the Uganda Cancer Institute during the study period.

The number of children lost to follow-up in our study (20, 22%) was slightly lower than that reported in a previous retrospective study (32%) of childhood cancer patients at the Uganda Cancer Institute by Mutyaba et al. [22]. This may be attributed to the recent introduction of a functional ocular oncology unit and personnel at UCI.

In bivariate analysis, the predictors of survival in our study were a history of eye swelling, a lag time of 7 months or more, extra ocular retinoblastoma in the affected eye(s), advanced disease stage (IIRC Group E) in the worst affected eye, completion of prescribed treatment, metastatic retinoblastoma, and bilateral enucleation. These predictors were similar to findings in a study in China by Gao et al., which showed that extraocular disease, treatment abandonment, bilateral disease, and advanced tumour stage were predictors of survival in children with retinoblastoma [23]. The above predictors indicate that most children with retinoblastoma seek medical attention at the advanced stages of disease and are referred to tertiary oncology centres with metastatic disease.

## 5. Conclusion

Children with retinoblastoma were referred to the Uganda Cancer Institute with advanced disease. In addition, the mortality of retinoblastoma patients at UCI was high, as evidenced by the very low 3-year survival. Thus, there is an urgent need for the early involvement of a multidisciplinary team of health workers in the management of retinoblastoma, including paediatric ophthalmologists, paediatric oncologists, ocular oncologists, radiation oncologists, and nurses, for better treatment outcomes.

## Figures and Tables

**Table 1:** Social demographic and clinical characteristics.

Variable	Frequency	Percentage (%)
Gender		
Female	38	42.2
Male	52	57.8
Age group at first sign in months		
0 to 12	40	40
13 to 36	29	32.2
Over 36	21	23.3
Age group at first UCI visit in months		
0 to 12	5	5.6
13 to 36	55	61.1
Over 36	30	33.3
Symptoms reported by parents		
Leukocoria	77	85.6
Eye reddening	58	64.4
Eye swelling	57	63.3
Jerky eye movements	8	8.9
Others	7	7.8
Family history of retinoblastoma	4	4.4
Had prior retinoblastoma treatment.	49	54.4
Enucleation	42	46.7
Radiotherapy	5	5.6
Chemotherapy	10	11.1
Lag time between first sign and UCI visit in months		
0 to 6	38	42.2
7 to 12	15	16.7
Above 12	37	41.1
Laterality		
Unilateral	65	72.3
Bilateral	25	27.8
Vision in the affected eye(s) at first UCI visit (*n* = 73 eyes)		
No	66	90.4
Yes	7	9.6
Clinical signs at first UCI visit		
Leukocoria	36	40
Proptosis	41	45.6
Enucleated with no visible tumour in socket	13	14.4
Disease extent		
Intraocular involvement only	27	30
Features of extraocular spread	63	70
Mean age at first UCI visit (months): overall	36.7 (±36.7)	
Unilateral	38.0 (±15.6)	
Bilateral	33.3 (±25.3)	

**Table 2:** IIRC classification of children with retinoblastoma at UCI based on worst affected eye(s).

IIRC group	Total (*N* = 90)	Bilateral Rb		Unilateral Rb	
		Frequency	% age	Frequency	% age
C	1 (1.1%)	0	0	1	1.5
D	16 (17.8%)	6	24	10	15.4
E	61 (67.8%)	16	64	45	69.2
Staging not done	12 (13.3%)	3	12	9	13.8

**Table 3:** Outcomes of children with retinoblastoma at the last follow-up contact.

Variable	Frequency (*N* = 90)	Percentage (%)
Metastatic spread		
No	46	51.1
Yes	44	48.9
Visual out-come		
Vision loss in one eye	64	71.1
Vision loss in both eyes	12	13.3
Sign of vision present	14	15.6
Eye ball loss		
No eye loss	38	42.2
Unilateral eye loss	49	54.4
Bilateral eye loss	3	3.3
Vital status		
Alive	37	41.1
Died	33	36.7
Lost to follow-up	20	22.2

**Table 4:** Bivariate and multivariate Cox regression of selected demographic and clinical characteristics for predictors of survival among children with retinoblastoma.

Variable	CHR (95% CI)	AHR (95% CI)	*P* value
Age group at first sign in months			
0 to 12	1	1	
13 to 36	1.08 (0.48 − 2.40)	0.89 (0.33 − 2.26)	0.772
Over 36	1.08 (0.45 − 2.58)	0.40 (0.14 − 1.13)	0.084
Sex: Male	1.04 (0.52 − 2.04)	1.12 (0.54 − 2.31)	0.769
Eye loss			
No	1	1	
1 eye enucleated	1.40 (0.67 − 2.94)	1.12 (0.51 − 2.45)	0.779
Both eyes enucleated	3.94 (1.64 − 9.46)	**3.53 (**1.33–9.36**)**	**0.011**
Metastatic spread			
No	1	**1**	
Yes	9.24 (3.88–22.02)	**8.93 (**3.23–24.69**)**	**0.001**
Lag time between first sign and UCI visit in months			
0 to 6	1	1	
7 and above	0.18 (0.07 − 0.42)	**0.10 (**0.04 − 0.25**)**	**0.001**
Proptosis			
No	1	1	
Yes	1.65 (0.82–3.31)	1.35 (0.52–3.48)	0.535
Eye swelling			
No	1		
Yes	2.62 (1.10–6.27)	**4.39 (**1.38–14.01**)**	**0.012**
Eye reddening			
No	1	1	
Yes	1.65 (0.73–3.70)	0.36 (0.14–0.94)	0.036

## Data Availability

Data is available on request through the author (email: kalibakaruth@gmail.com).

## References

[1] Boubacar T., Fatou S., Fousseyni T. (2010). a 30-month prospective study on the treatment of retinoblastoma in the Gabriel Toure teaching hospital, Bamako, Mali. *British Journal of Ophthalmology*.

[2] Dimaras H., Kimani K., Dimba E. A. O. (2012). Retinoblastoma. *The Lancet*.

[3] Fabian I. D., Abdallah E., Abdullahi S. U. (2020). Global retinoblastoma presentation and analysis by National Income Level. *JAMA Oncology*.

[4] Aerts I., Lumbroso-Le Rouic L., Gauthier-Villars M., Brisse H., Doz F., Desjardins L. (2006). Retinoblastoma. *Orphanet Journal of Rare Diseases*.

[5] Shields C. L., Shields J. A. (2004). Diagnosis and management of retinoblastoma. *Cancer Control*.

[6] Olisa E. G., Chandra R., Jackson M. A., Kennedy J., Williams A. O. (1975). Malignant tumors in American black and Nigerian children: a comparative study. *Journal of the National Cancer Institute*.

[7] Fabian I. D., Stacey A. W., Foster A. (2021). Travel burden and clinical presentation of retinoblastoma: analysis of 1024 patients from 43 African countries and 518 patients from 40 European countries. *The British Journal of Ophthalmology*.

[8] Chawla B., Hasan F., Azad R. (2016). Clinical presentation and survival of retinoblastoma in Indian children. *British Journal of Ophthalmology*.

[9] Kazadi Lukusa A., Aloni M. N., Kadima-Tshimanga B. (2012). Retinoblastoma in the democratic republic of Congo: 20-year review from a tertiary hospital in Kinshasa. *Journal of Cancer Epidemiology*.

[10] Waddell K. M., Kagame K., Ndamira A. (2015). Clinical features and survival among children with retinoblastoma in Uganda. *The British Journal of Ophthalmology*.

[11] Li S.-Y., Chen S. C.-C., Tsai C.-F., Sheu S.-M., Yeh J.-J., Tsai C.-B. (2016). Incidence and survival of retinoblastoma in Taiwan: a nationwide population-based study 1998–2011. *British Journal of Ophthalmology*.

[12] Ali A. A., Elsheikh S. M., Elhaj A. (2011). Clinical presentation and outcome of retinoblastoma among children treated at the National Cancer Institute (NCI) in Gezira, Sudan: a single institution experience. *Ophthalmic Genetics*.

[13] Lavaju P., Badhu B., Shah S., Chaudhary S., Upadhyaya P. (2017). Clinical profile of retinoblastoma presenting in a tertiary care hospital, eastern Nepal. *Health Renaissance*.

[14] Kruger M., Reynders D., Omar F., Schoeman J., Wedi O., Harvey J. (2014). Retinoblastoma outcome at a single institution in South Africa. *South African Medical Journal*.

[15] Nyamori J. M., Kimani K., Njuguna M. W., Dimaras H. (2012). The incidence and distribution of retinoblastoma in Kenya. *British Journal of Ophthalmology*.

[16] Nabie R., Taheri N., Fard A. M., Fouladi R. F. (2012). Characteristics and clinical presentations of pediatric retinoblastoma in North-Western Iran. *International Journal of Ophthalmology*.

[17] Özkan A., Pazarli H., Celkan T. (2006). Retinoblastoma in Turkey: survival and clinical characteristics 1981–2004. *Pediatrics International*.

[18] Lim F. P. M., Soh S. Y., Iyer J. V., Tan A. M., Swati H., Quah B. L. (2013). Clinical profile, management, and outcome of retinoblastoma in Singapore. *Journal of Pediatric Ophthalmology and Strabismus*.

[19] Tamboli D., Topham A., Singh N., Singh A. D. (2015). Retinoblastoma: a SEER dataset evaluation for treatment patterns, survival, and second malignant neoplasms. *American Journal of Ophthalmology*.

[20] Kao L.-Y., Su W.-W., Lin Y.-W. (2002). Retinoblastoma in Taiwan: survival and clinical characteristics 1978-2000. *Japanese Journal of Ophthalmology*.

[21] Naseripour M., Nazari H., Bakhtiari P., Modarres-zadeh M., Vosough P., Ausari M. (2009). Retinoblastoma in Iran: outcomes in terms of patients' survival and globe survival. *The British Journal of Ophthalmology*.

[22] Mutyaba I., Wabinga H. R., Orem J., Casper C., Phipps W. (2019). Presentation and outcomes of childhood cancer patients at Uganda Cancer Institute. *Global Pediatric Health*.

[23] Gao J., Zeng J., Guo B. (2016). Clinical presentation and treatment outcome of retinoblastoma in children of South Western China. *Medicine*.

